# Senescent Cells Impair Erectile Function through Induction of Endothelial Dysfunction and Nerve Injury in Mice

**DOI:** 10.1371/journal.pone.0124129

**Published:** 2015-04-20

**Authors:** Hiroaki Nishimatsu, Etsu Suzuki, Yasuho Saito, Aya Niimi, Akira Nomiya, Hiroshi Fukuhara, Haruki Kume, Yukio Homma

**Affiliations:** 1 The Department of Urology, Faculty of Medicine, University of Tokyo, Bunkyo-ku, Tokyo, Japan; 2 Institute of Medical Science, St. Marianna University School of Medicine, Miyamae-ku, Kawasaki, Japan; Max-Delbrück Center for Molecular Medicine (MDC), GERMANY

## Abstract

Erectile dysfunction (ED) is a major health problem, particularly in the elderly population, which is rapidly increasing. It is necessary to elucidate the mechanism by which ED occurs in the elderly. Cellular senescence is commonly detected in old tissues, and it is well known that senescent cells not only withdraw from the cell cycle but also remain viable and actively produce a variety of cytokines. We examined the effect of senescent cells on erectile function after injection of senescent cells into the penises of mice. Human umbilical vein endothelial cells were infected with an adenovirus expressing a constitutively active mutant of Ras to induce senescence, and were injected into the penises of nude mice. These senescent cells expressed proinflammatory cytokines such as interleukin-1β (IL-1β). Injection of senescent cells impaired erectile function, as assessed by the measurement of intracavernous pressure. Although the structure of the cavernous body did not remarkably change, expression of the catalytically active form of endothelial nitric oxide synthase and that of total neural nitric oxide synthase significantly decreased after injection. The penises injected with the senescent cells expressed human IL-1β and subsequently endogenous proinflammatory cytokines such as mouse IL-1β and tumor necrosis factor-α. These results suggested that senescent cells impaired erectile function through induction of endothelial dysfunction and nerve injury. These effects may be mediated by proinflammatory cytokines produced by senescent cells.

## Introduction

Erectile dysfunction (ED) is a major global health concern and affects more than 10 million Japanese males [1]. It is estimated that more than 50% Japanese males who are aged >60 years suffer from ED. There are several reasons why ED frequently occurs in the elderly population. First, hypogonadism occurs in the elderly population, and it has been reported that testosterone replacement therapy restores erectile function in these patients [2, 3]. Second, the elderly often suffer from diabetes, hypertension, and hyperlipidemia, all of which potentially induce vascular dysfunction in the penis [4, 5]. Third and the main issue of this paper is that morphological and/or functional changes may occur in the cells of the cavernous body when the cells “live” for long periods or they are persistently exposed to stresses, even in the absence of hypogonadism or metabolic disorders [6].

One of the characteristic changes observed in the old tissues at cellular level is cellular senescence. Cellular senescence was first described by Hayflick. He observed that primary cultured cells that had been explanted from tissues did not proliferate indefinitely and eventually exited the cell cycle. He hypothesized that the finite lifecycle of these cells may be an expression of aging or senescence at the cellular level [7]. Therefore, it is well established that cellular senescence represents a stable and long-term cell cycle arrest, even though cells remain viable and metabolically active. Senescent cells produce a variety of cytokines and chemokines, which may explain why they cause inflammation in the surrounding tissues [8]. We have recently demonstrated that human umbilical vein endothelial cells (HUVECs) produce cytokines such as interleukin-1β (IL-1β) when senescence was induced in HUVECs [9]. Although telomere shortening is a major cause of cellular senescence (known as replicative cellular senescence) [10], other stimuli, such as the activation of oncogenes and oxidative stress, can also cause cellular senescence (termed premature cellular senescence) [11, 12]. Cellular senescence reportedly occurs in the blood vessels of diabetic animals [13, 14], probably because oxidative stress increases during diabetes ([15, 16].

Because senescent cells potentially induce inflammation in the surrounding tissues, it is possible that, in the penis, these cells are implicated in the pathogenesis of ED in the elderly and in diabetic patients. However, little is known as to whether senescence occurs in the penis. Even if senescence does occur in the penis, it is difficult to analyze the effect of senescent cells on erectile function. This is because inflammation and subsequent tissue damage simultaneously occur, which may not be mediated by senescent cells. For example, vascular injury may occur in diabetes directly through inflammation caused by hyperglycemia [17] and adipocytokines such as tumor necrosis factor-α (TNF-α) [18] as well as through inflammation caused by senescent cells. Vascular structures of the penis in the elderly may be injured by coexisting metabolic disorders [19] as well as by inflammation caused by senescent cells. Thus, the presence of senescent cells in the penis *per se* does not always indicate their significant roles in ED.

In this study using diabetic mice, we examined whether senescence occurs in the penis. To study the direct effects of senescent cells on erectile function, we also injected senescent cells into penises of nude mice and examined whether senescent cells affect erectile function.

## Materials and Methods

### Cell culture

HUVECs were purchased from Sanko Junyaku Co., Ltd. (Tokyo, Japan) and cultured in HuMedia-EG2 (Kurabo, Osaka, Japan).

### Adenovirus infection

Infection with adenovirus that expresses a constitutively active mutant of mouse Ras (AdRas12V) has been previously reported [20]. Adenovirus that expresses green fluorescence protein (AdGFP) was obtained from Quantum Biotechnologies (Montreal, Canada). HUVECs were infected with these adenoviruses at a multiplicity of infection (MOI) of 20, and subsequently cultured in HuMedia-EG2 for 7 days to induce senescence.

### Animal study

All animal protocols were approved by the institutional committee for animal research, Tokyo University, Japan. Seven week-old male C57BL/6J mice and nude mice were purchased from Charles River (Yokohama, Japan). Streptozotocin (STZ; 250 mg/kg body weight; Sigma-Aldrich) was dissolved in citrate buffer (pH 4.5) and injected into the peritoneal space of C57BL/6J mice. Blood glucose level was measured 4 weeks later to confirm that these mice had become diabetic. Nude mice were not injected with STZ and therefore were not diabetic. Single cell suspension of HUVECs (50,000 cells) was diluted in saline up to 50 μL and injected into the glans of the penis of nude mice. Intracavernous pressure (ICP) measurement was performed 2, 4, and 6 weeks after injection. Following euthanasia by cervical dislocation, the penis was also harvested for histochemical analysis and Western blot analysis at the above mentioned time points. ICP measurement was performed in the same manner as previously reported, with slight modifications [21]. Mice were anesthetized with intraperitoneal injection of ketamine (100 mg/kg body weight) and xylazine hydrochloride (10 mg/kg body weight). The right carotid artery was cannulated with a PE-10 polyethylene tube to continuously monitor mean arterial pressure (MAP). The penis was denuded of overlying skin and cannulated with a heparinized 27-gauge needle connected to a pressure transducer to continuously monitor ICP. The pelvic and cavernous nerves were isolated following a lower midline abdominal incision. The right cavernous nerve was then stimulated with a stainless platinum bipolar hook electrode (Unique Medical Co., Tokyo) connected to a square wave stimulator (Nihon Kohden Co., Tokyo). The stimulation parameters were as follows: stimulation duration, 5 s; voltage, 5.0 V; pulse duration, 2 ms; and stimulation frequency, 20 Hz. Area under the curve (AUC) of ICP traces and the ratio of peak ICP to MAP (ICP/MAP) were used to evaluate erectile function. AUC was measured from the point at which ICP started to increase from base line values after electrical stimulation to the point at which ICP decreased and reached a plateau. ICP x time was integrated every 0.2 s to calculate AUC.

### SA-β-Gal assay

After perfusing the penis with saline, frozen sections of the penis (cross sections; 10 μm each) were prepared. The penis was subsequently fixed in 4% paraformaldehyde for 10 min, and senescence-associated-β-galactosidase (SA-β-Gal) staining was performed using a cellular senescence detection kit (Cell Biolabs, Inc., San Diego, CA) according to the manufacturer’s protocol.

### Histochemistry

The penis was fixed using perfusion with 4% paraformaldehyde. It was then embedded in paraffin and sliced into 5-μm cross sections that were deparaffinized, rehydrated, and subjected to the Elastica van Gieson stain. Elastica van Gieson stain was performed by a standard method. In brief, the specimens were consecutively stained with resorcin‐fuchsin staining solution (Muto Pure Chemicals, CO., LTD., Tokyo, Japan), hematoxylin staining solution, and van Gieson staining solution (Muto Pure Chemicals, CO., LTD., Tokyo, Japan). For immunohistochemistry, sections were incubated with a primary antibody reactive to neural nitric oxide synthase (nNOS: 1:400, Abcam, ab76067) and heterochromatin protein-1γ (HP-1γ: 1:400, Abcam, ab66617). Sections were subsequently incubated with biotinylated secondary antibody and finally horseradish peroxidase-labeled streptavidin according to the instructions provided by the manufacturer (DAKO, Cambridgeshire, UK).

### Protein extraction and Western blot analysis

Total protein content was extracted from penises homogenized in cell lysis buffer (50 mM Tris-HCl (pH 8.0), 150mM NaCl, 1% NP-40) containing 2 μg/mL aprotinin, 2 μg/mL leupeptin and 1 mM phenylmethylsulfonyl fluoride. Western blot analysis was performed as previously described [22]. VE-cadherin (VE-Cad), α-smooth muscle actin (SMA), phospho-endothelial nitric oxide synthase (eNOS), total eNOS, and nNOS expressions were normalized by calculating the ratio of the expression of those proteins to that of β-actin. The dilutions of the antibodies used in this study were as follows. Anti-VE-Cad antibody: 1:200, Santa-Cruz, sc-9989; anti-SMA antibody: 1:200, Santa-Cruz, sc-130617; anti-phospho-eNOS antibody: 1:500, Cell Signaling, 9571; anti-eNOS antibody: 1:500, Cell Signaling, 9572; anti-nNOS antibody: 1:250, LifeSpan BioSciences, LS-C37730; anti-β-actin antibody: 1:300, Santa-Cruz, sc-47778; anti-p21^CIP1^ antibody: 1:200, Santa-Cruz, sc-397; anti-p53 antibody: 1:500, Abcam, ab26.

### RNA extraction and real time PCR

HUVECs were injected into the penises of nude mice and RNA was extracted from the penises 1, 7, 14 and 28 days later. To extract total RNA, the penises were homogenized using TRIZOL Reagent (Life Technologies, Tokyo, Japan). Reverse transcription of total RNA was performed using ReverTra Ace qPCR RT Master Mix (TOYOBO, Osaka, Japan). The expression of IL-1β, interleukin-6 (IL-6), TNF-α, and glyceraldehyde 3-phosphate dehydrogenase (GAPDH) was examined by real time PCR using an SYBR Green dye (Thunderbird SYBR qPCR Mix, TOYOBO, Japan). Primers used were as follows:

HumanIL-1β sense: 5’-CGAATCTCCGACCACCACTAC-3’

HumanIL-1β antisense: 5’-TCCATGGCCACAACAACTGA-3’

MouseIL-1β sense: 5’-AGGCAGGCAGTATCACTCATTGT-3’

MouseIL-1β antisense: 5’-GGAAGGTCCACGGGAAAGAC-3’

MouseIL-6 sense: 5’-ACAAGTCGGAGGCTTAATTACACAT-3’

MouseIL-6 antisense: 5’-TTGCCATTGCACAACTCTTTTC-3’

MouseTNF-α sense: 5’-TGATCCGCGACGTGGAA-3’

MouseTNF-α antisense: 5’-CCGCCTGGAGTTCTGGAA-3’

MouseGAPDH sense: 5’-TGTGTCCGTCGTGGATCTGA-3’

MouseGAPDH antisense: 5’-ACCACCTTCTTGATGTCATCATACTT-3’

### Statistical analysis

All values were expressed as the mean ± standard error of the mean (SEM). Statistical analyses were performed using analysis of variance followed by the Student-Newman-Keuls test. Differences with a P value of <0.05 were considered statistically significant.

## Results

### Senescence occurs in the penises of diabetic mice

**Fig 1:**
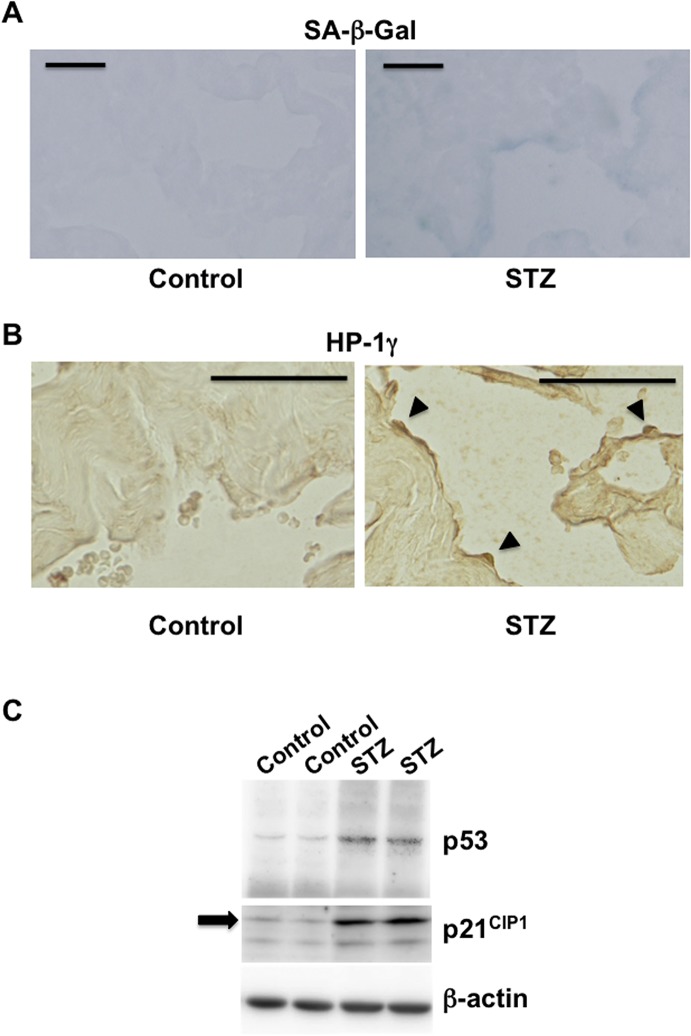
Senescence occurs in the penis of diabetic mice. A) The penises were isolated from STZ-induced diabetic C57BL/6J mice 7 weeks after STZ injection. Frozen penile sections were subjected to SA-β-Gal staining. The penises of age-matched control mice were also used. Scale bars = 50 μmeter. B) Immunohistochemical analysis of the penises isolated from STZ-induced diabetic C57BL/6J mice and age-matched control mice. HP-1γ was stained. Arrows indicate the positively stained area. Scale bars = 50 μmeter. C) Western blot analysis of senescence-associated markers. Proteins were extracted from the penises of STZ-induced diabetic C57BL/6J mice (STZ) 7 weeks after STZ injection. Proteins were also extracted from the penises of age-matched control mice (Control). p53 and p21^CIP1^ expressions were examined. The arrow indicates the bands corresponding to the size of p21^CIP1^.

We first measured the casual blood glucose level of the non-diabetic control C57BL/6J mice and STZ-induced diabetic C57BL/6J mice to confirm that these mice became diabetic (Blood glucose level: control; 165.8±15.5 mg/dL vs. STZ; 364.7±28.3 mg/dL, n = 7 per group, P<0.001). We next examined whether cellular senescence occurs in the cavernous body of diabetic mice. The penis of STZ-induced diabetic C57BL/6J mice was sporadically stained with SA-β-Gal (Fig 1A). The positive area was predominantly located on the surface of the trabeculae of the cavernous body corresponding to the vascular endothelial cells (VECs) and/or vascular smooth muscle cells (VSMCs). SA-β-Gal staining was well correlated with HP-1γ immunostaining [23]. Therefore, we performed HP-1γ staining (Fig 1B). HP-1γ was also sporadically stained on the surface of the trabeculae, corresponding to the area of the endothelium. In accordance with this result, expression of p53 and p21^CIP1^ remarkably increased in diabetic C57BL/6J mice compared with non-diabetic control mice (Fig 1C). These results suggested that cellular senescence occurs in the cavernous body of diabetic mice.

### Injection of senescent cells into the penis impairs erectile function

**Fig 2:**
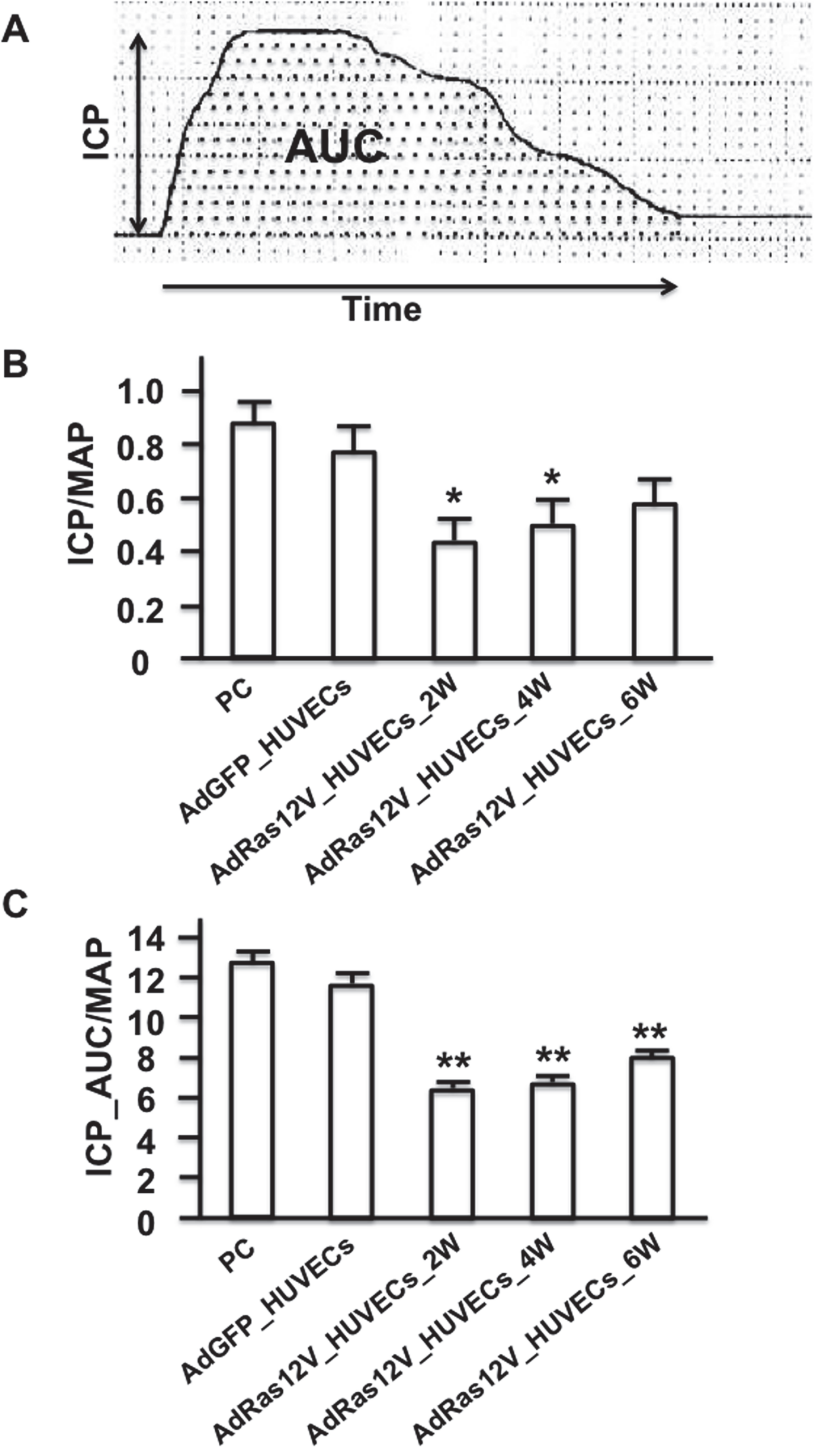
Injection of senescent cells in the penis impairs erectile function. HUVECs were infected with AdRas12V (AdRas12V_HUVECs) to induce cellular senescence. HUVECs were also infected with AdGFP (AdGFP_HUVECs) as the control. These cells were injected into the cavernous body of nude mice, and ICP and MAP were measured 2 (2W), 4 (4W) and 6 (6W) weeks after injection. ICP data obtained from control nude mice that were not injected with the cells and from nude mice 2 weeks after injection with AdGFP_HUVECs were used as the positive control. A) Schematic representation of ICP traces. The dotted area represents AUC. B) Bar graphs comparing ICP/MAP among the groups (n = 5 per group). *: P<0.05 vs. the AdGFP_HUVECs injection. C) Bar graphs comparing ICP_AUC/MAP among the groups (n = 5 per group). **: P<0.001 vs. the AdGFP_HUVECs injection.

To examine the function of senescent cells, we decided to inject senescent HUVECs into the cavernous body of nude mice. Nude mice [24] were used in this study to avoid inflammation and immunological rejection due to injection of foreign cells (human cells) into mice [25]. HUVECs were infected with AdRas12V to induce cellular senescence as previously reported [9]. HUVECs infected with AdGFP (AdGFP_HUVECs) or AdRas12V (AdRas12V_HUVECs) were injected into the penises of nude mice and intracavernous pressure (ICP) was measured 2, 4, and 6 weeks after injection. ICP/mean arterial pressure (MAP) significantly decreased 2 and 4 weeks after AdRas12V_HUVECs injection compared with AdGFP_HUVECs injection (Fig 2). ICP/MAP started to recover 6 weeks after the AdRas12V_HUVECs injection, and no statistically significant difference was observed between AdGFP_HUVECs-injected and AdRas12V_HUVECs-injected groups at this time point. The AUC of ICP traces/MAP (ICP_AUC/MAP) also significantly decreased 2 weeks after the AdRas12V_HUVECs injection compared with the AdGFP_HUVECs injection, and this significant decrease continued until 6 weeks after injection. Overall, ICP/MAP and ICP_AUC/MAP most strikingly decreased 2 weeks after the AdRas12V_HUVECs injection, and started to recover after that time point.

### Histology of penis

**Fig 3:**
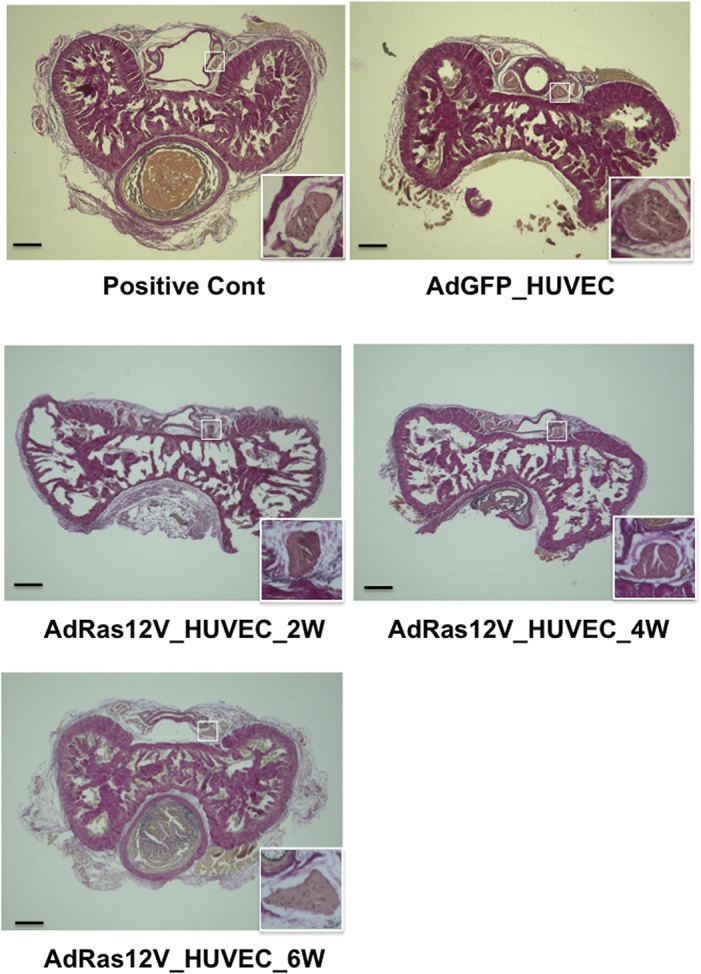
Elastica van Gieson staining of the cavernous body isolated from age-matched nude mice and nude mice injected with AdGFP_HUVECs or AdRas12V_HUVECs. The penises were isolated 2 weeks after AdGFP_HUVECs injection, and 2 (2W), 4 (4W), and 6 (6W) weeks after the AdRas12V_HUVECs injection. The penises were also isolated from nude mice that were not injected with HUVECs as the positive control (Positive Cont). The boxed areas corresponding to the dorsal penile nerve are enlarged and shown at the right lower corners. Scale bar = 200 μmeter.

**Fig 4:**
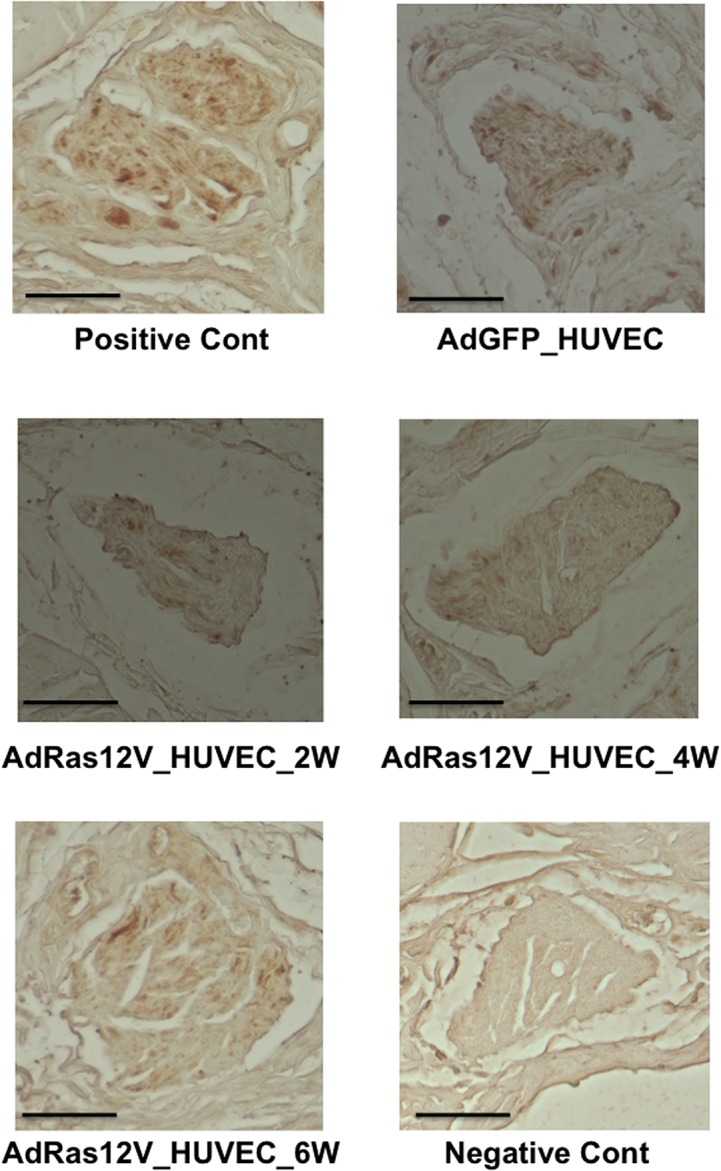
Immunohistochemical staining of nNOS in the dorsal penile nerve. Experiments were performed in the same way as described in the legend for Fig 3. nNOS was stained. The photo of the negative control (Negative Cont) is also shown in which the incubation with primary antibody against nNOS was omitted. Scale bar = 40 μmeter.

We subsequently analyzed the penis morphology using Elastica van Gieson stain. When AdGFP_HUVECs were injected into the penis, the morphology of the cavernous body did not remarkably change compared with the positive control penis that was not injected with HUVECs (Fig 3). When AdRas12V_HUVECs were injected into the penis, the morphology of the cavernous body was also conserved. The size of each trabecula did not remarkably change after cell injection. This is in marked contrast to the penis of diabetic rats, in which the size of each trabecula became smaller than that of the penis of non-diabetic rat [21]. Morphology of the dorsal penile nerve was also examined. The morphology did not remarkably change when AdRas12V_HUVECs were injected into the penis. However, because it has been established that nitric oxide (NO) released from nitrergic nerves [26], which express nNOS and release NO as a cotransmitter with acetylcholine, play pivotal roles particularly in the initiation of erection [27], we examined nNOS expression by immunohistochemical analysis (Fig 4). nNOS was positively stained in the dorsal penile nerve in both the positive control and the AdGFP_HUVECs-injected penises. When AdRas12V_HUVECs were injected into the penis, nNOS staining was remarkably decreased at 2 and 4 weeks after injection. nNOS staining appeared to be recovering at 6 weeks after injection.

### Protein expression

**Fig 5:**
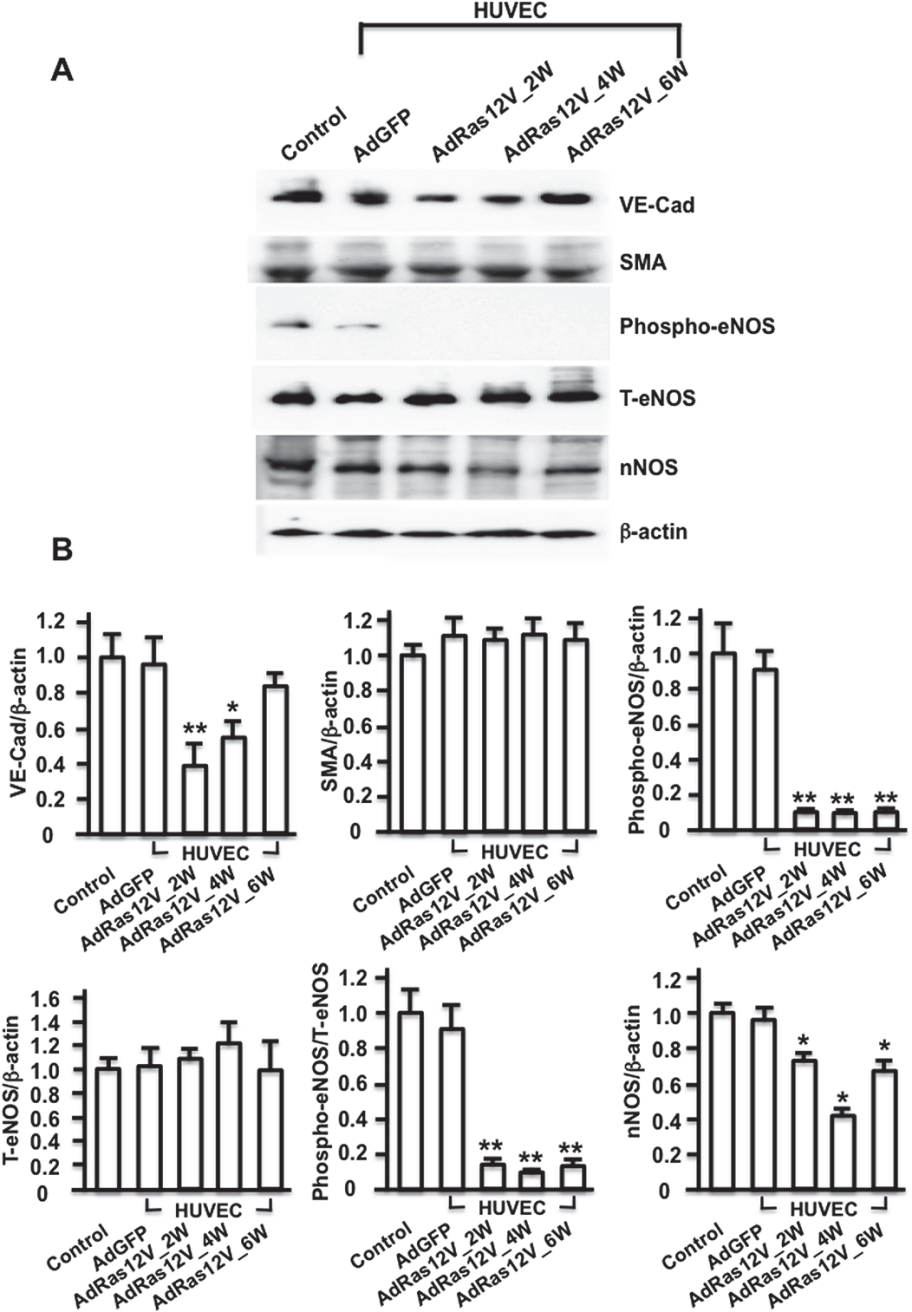
Western blot analysis of VE-Cad, SMA, phospho-eNOS, total eNOS, and nNOS expressions. A) The penises were isolated for protein extraction 2 weeks after the AdGFP_HUVECs injection, and 2 (2W), 4 (4W), and 6 (6W) weeks after the AdRas12V_HUVECs injection. Proteins were also extracted from age-matched control mice (Control). Representative photographs are shown. B) Histograms showing the relative intensity of the bands (n = 4 per group). * and **: P<0.05 and P<0.01, respectively vs. AdGFP_HUVECs injection. T-eNOS: total eNOS.

To quantify the markers for blood vessels and nitrergic nerves, Western blot analysis was performed (Fig 5). VE-Cad expression significantly decreased at 2 and 4 weeks after AdRas12V_HUVECs injection compared with AdGFP_HUVECs injection. Its expression recovered 6 weeks after AdRas12V_HUVECs injection. In contrast, SMA and total eNOS expressions did not significantly change after AdRas12V_HUVECs injection. These results were in marked contrast to those observed in the penises of diabetic rats, where SMA expression significantly decreased compared with that in non-diabetic control rats [21, 28]. Interestingly, the expression of phospho-eNOS, which is phosphorylated at Ser1177 and catalytically active, remained significantly low at 2, 4, and 6 weeks after the AdRas12V_HUVECs injection compared with that after the AdGFP_HUVECs injection. The ratio of phospho-eNOS to total eNOS also remained significantly low in the AdRas12V_HUVECs-injected group compared with that in the AdGFP_HUVECs-injected group. These results suggested that although the vascular structure in the penis was relatively conserved after injection of AdRas12V_HUVECs, endothelial dysfunction occurred. nNOS expression significantly decreased at 2 and 4 weeks after the AdRas12V_HUVECs injection, and the expression remained low 6 weeks later compared with that after the AdGFP_HUVECs injection, although the expression was recovering.

### Expression of proinflammatory cytokines in the penis

**Fig 6:**
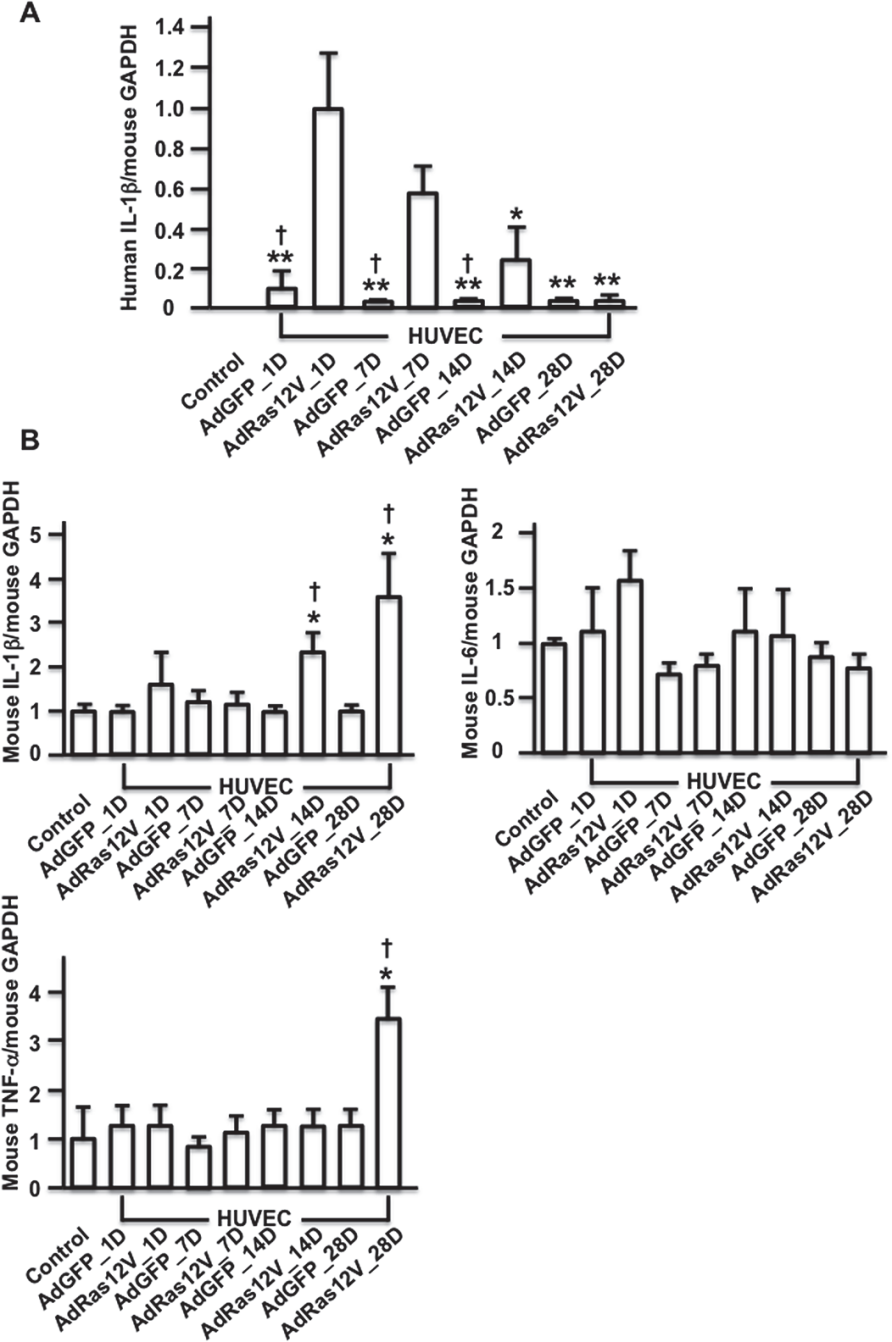
The expression of proinflammatory cytokines in the penis. AdGFP_HUVECs (AdGFP) and AdRas12V_HUVECs (AdRas12V) were injected in the penises of nude mice and RNA was extracted from the penises 1 (1D), 7 (7D), 14 (14D), and 28 (28D) days after injection for real time PCR analysis. Nontreated mice (Control) were also analyzed. A) The expression of human IL-1β that was produced from injected HUVECs. The ratio of human IL-1β to mouse GAPDH was calculated to demonstrate human IL-1β expression in the penises of nude mice. The expression of human IL-1β in AdRas12V_HUVECs–injected penises on day 1 was calculated as 1.0 and the fold induction was shown in other groups (n = 5 per group). * and **: P<0.05 and P<0.01, respectively vs. AdRas12V_HUVECs–injected penises on day 1, †: P<0.01 vs. AdRas12V_HUVECs–injected penises at each time point. B) The expression of mouse IL-1β, mouse IL-6, and mouse TNF-α in the AdGFP_HUVECs- or AdRas12V_HUVECs-injected penises. The expression of these cytokines in nontreated control mice (Control) was calculated as 1.0 and fold induction was shown in other groups (n = 5 per group). *: P<0.05 vs. Control, †: P<0.05 vs. AdGFP_HUVECs–injected penises at each time point.

To analyze how long injected HUVECs remained in the penis and produced proinflammatory cytokines, AdGFP_HUVECs and AdRas12V_HUVECs were injected into the penises of nude mice, and real time PCR analysis was performed. We also analyzed the production of endogenous proinflammatory cytokines. The expression of human IL-1β was below the detectable levels in control nude mice into which HUVECs were not injected. The expression of human IL-1β was maximal in the AdRas12V_HUVECs-injected penis 1 day after injection, and the expression had gradually decreased by 14 days after injection. The expression of human IL-1β had almost disappeared 28 days after injection. The expression of human IL-1β in AdGFP_HUVECs-injected penises was significantly lower than that in AdRas12V_HUVECs-injected penises (Fig 6A). We also analyzed the expression of mouse IL-1β, IL-6, and TNF-α to detect the expression of endogenous proinflammatory cytokines (Fig 6B). The expression of mouse IL-1β in AdRas12V_HUVECs-injected penises did not change significantly compared with that in the control nude mice until 7 days after injection. However, the expression of mouse IL-1β increased significantly in AdRas12V_HUVECs-injected penises compared with that in the control nude mice 14 and 28 days after injection. The expression of mouse IL-1β in AdRas12V_HUVECs-injected penises was also significantly higher than that in AdGFP_HUVECs-injected penises 14 and 28 days after injection. The expression of IL-6 did not change significantly during the time course. The expression of mouse TNF-α was significantly higher in AdRas12V_HUVECs-injected penises than that in the control mice 28 days after injection. These results suggested that, although the production of proinflammatory cytokines from injected HUVECs ceased within 14 days, the production of endogenous proinflammatory cytokines started and maintained inflammation thereafter.

## Discussion

In this study, we demonstrated that senescence occurred in the penis of diabetic mice. We also showed that injection of senescent cells into the penis impaired erectile function probably because senescent cells induced endothelial dysfunction and nerve injury. It has recently been shown that human atherosclerotic plaques contain SA-β-Gal positive VECs and VSMCs that exhibit the morphological features of senescence [29, 30]. It has also been shown that blood vessels of diabetic animals contain SA-β-Gal positive VECs [13, 14]. Therefore, senescence appears to commonly occur in the blood vessels.

One of the most important problems in the study of senescence is that although senescence can be induced in a variety of diseases, it is difficult to show that the observed functional and morphological changes in the organs are caused solely by senescence. This is because several signaling pathways that may not be related to the induction of senescence are also simultaneously activated in diseased states. For example, in the diabetic state, hyperglycemia and a variety of adipocytokines secreted from visceral adipose tissue are implicated in the induction of vascular inflammation [31–33]. These factors may cause inflammation in the organs, independent of the induction of senescence. Thus, the organ damage observed in diabetes is caused not only by senescence but also by other stimuli that induce inflammation in the organs. To clearly examine whether senescent cells affect erectile function, we injected senescent HUVECs into the penis of nude mice and demonstrated that the senescent cells impaired erectile function.

ICP seemed to transiently decrease after injection of senescent HUVECs, and ICP started to recover 4 weeks after injection, although ICP did not completely return to the control level even at 6 weeks after injection. To examine the mechanism, we performed histological and Western blot analyses. Although the morphology of the cavernous body and the dorsal penile nerve did not remarkably change after injection of senescent HUVECs, VE-Cad expression transiently decreased. Furthermore, phospho-eNOS expression significantly decreased, and it remained decreased for 6 weeks. nNOS expression also significantly decreased until 4 weeks after the injection of senescent cells and started to increase thereafter. These results suggested that the injection of senescent cells induced endothelial dysfunction and nerve injury. Interestingly, although the production of human IL-1β almost disappeared within 14 days, the penises injected with senescent HUVECs produced endogenous mouse IL-1β and TNF-α thereafter. These results suggested that senescent cells secreted proinflammatory cytokines in a paracrine manner and that these cytokines in turn stimulated the production of proinflammatory cytokines in the surrounding tissues, which contributed to the continued inflammation after the cessation of cytokine production from senescent cells. It is well established that senescent cells undergo massive changes in gene expression, and actively produce a variety of proinflammatory cytokines and immune modulators. These changes have been referred to be the senescence-associated secretory phenotype [34]. We have recently demonstrated that AdRas12V-infected HUVECs produce proinflammatory cytokines such as IL-1β [9]. IL-1β is a potent proinflammatory cytokine that stimulates the expression of adhesion molecules on endothelial cells [35] and is frequently expressed in human atherosclerotic plaques [29]. In addition, IL-1βdeficiency decreases the severity of atherosclerosis in apolipoprotein E knockout mice [36]. TNF-α is also a potent proinflammatory cytokine that stimulates the expression of adhesion molecules and matrix metalloproteinases [37, 38]. TNF-α deficiency also reduced atherosclerosis in apolipoprotein E knockout mice [39]. Therefore, both IL-1β and TNF-α induce vascular inflammation and potentially impair erectile function.

Although both VE-Cad and eNOS are expressed in VECs, total eNOS expression was conserved after injection of senescent cells in contrast to a decrease in VE-Cad expression. We have demonstrated VE-Cad, total eNOS and, SMA expressions significantly decreased in the penises of diabetic rats, where the morphology of the cavernous body remarkably changed: the trabeculae of the cavernous body became smaller than those in non-diabetic control rats [21, 40]. Total eNOS expression may be maintained in relatively “mild” vascular injury. A similar phenomenon was demonstrated in a previous report, in which total eNOS expression was conserved in the penis of rats fed a high fat diet, although the endothelial area was significantly decreased in the cavernous body [41]. It was also reported that the SMA content did not decrease in a rat model of hyperlipidemia, although the endothelial area significantly decreased [42], suggesting that SMA expression is also maintained in relatively “mild” vascular injury.

### Study limitations

Diabetic mice were used to demonstrate cellular senescence because it is faster to establish senescence in diabetic mice than that in aged mice. However, the analysis of aged mice will also be required in the future. We analyzed the transient effects of the injection of senescent cells on erectile function. It is also desirable to analyze the chronic effect of senescent cells on erectile function. Usage of transgenic mice expressing Ras12V in VECs in an inducible manner will be a promising strategy for future analysis of this point.

## Conclusions

Cellular senescence occurs in the penis. Senescent cells potentially induce erectile dysfunction through induction of endothelial dysfunction and nerve injury, probably because senescent cells produce proinflammatory cytokines that cause inflammation in the penis. Regulation of cellular senescence will be useful in treating elderly and diabetic patients who suffer from ED.
